# Creative Argumentation: When and Why People Commit the Metaphoric Fallacy

**DOI:** 10.3389/fpsyg.2018.01815

**Published:** 2018-09-25

**Authors:** Francesca Ervas, Antonio Ledda, Amitash Ojha, Giuseppe Antonio Pierro, Bipin Indurkhya

**Affiliations:** ^1^Department of Pedagogy, Psychology, Philosophy, University of Cagliari, Cagliari, Italy; ^2^Department of Mathematics and Informatics, University of Cagliari, Cagliari, Italy; ^3^Department of Philosophy, Jagiellonian University, Kraków, Poland

**Keywords:** metaphor, analogy, creativity in argumentation, lexical ambiguity fallacy, belief in the conclusion

## Abstract

This article aims to understand when and why people accept fallacious arguments featuring metaphors (metaphoric fallacy) as sound arguments. Two experiments were designed to investigate, respectively, when and why participants fell into the metaphoric fallacy. In the first experiment, participants were provided with a series of syllogisms, presented in natural language, containing in their first premise either a lexically ambiguous, literal middle term or a metaphorical middle term, i.e. the term that “bridges” the first premise with the second premise, and ending with a true, false or plausible conclusion. For each argument they were asked to evaluate whether the conclusion followed from the premises. Results show that the metaphoric fallacy is harder to detect in case of arguments with plausible conclusion with a conventional metaphor rather than a novel metaphor as middle term. The second experiment investigated why participants considered the metaphoric fallacy with plausible conclusion as a strong argument. Results suggest that participants’ belief in the conclusion of the argument, independent from the premises, is a predictor for committing the metaphoric fallacy. We argue that a creative search for alternative reasons justifies participants’ falling into the metaphoric fallacy, especially when the framing effect of a metaphor covertly influences the overall reading of the argument. Thus, far from being a source of irrationality, metaphors might elicit a different style of reasoning in argumentation, forcing participants to find an alternative interpretation of the premises that guarantees the believed conclusion. In this process, conventional metaphors are revitalized and extended through the second premise to the conclusion, thereby entailing an overall metaphorical reading of the argument.

## Introduction

One of the aims of argumentation theory is to provide a satisfactory explanation of how people evaluate arguments rationally according to specific norms and standards (Van Eemeren and Grootendorst, 2004; Tindale, 2006; Walton, 2006). Reasoning errors might shed light on explaining how argumentative rationality moves away from norms and standards (Woods and Walton, 1989; Hamblin, 1970). In this perspective, it may happen to discover that laypeople are often not rational in evaluating arguments, thus breaking normative ties and systematically falling into argumentation fallacies (Van Eemeren, 1992; Ariely, 2008; Walton, 2010). As pointed out by Jonathan Evans (2014), this does not mean that agents are (completely) irrational, as often claimed in psychology of reasoning and decision making (Wason, 1966; Wason and Johnson-Laird, 1972). This work attempts to suggest that they might simply be creative in argumentation, finding alternative reasons to make sense of their own conclusions and evaluations. For example, when someone commits a reasoning error, this might be due to the intuitive search for alternative reasons, especially in the case of non-literal language used in argumentation. Here, the term “argumentation" is used in an intuitive sense, as covering any conveying of alleged reasons in support of conclusions that a speaker wishes the interlocutor to draw from some premises. In this intuitive sense, argumentation is strictly bound to reasoning as the activity of evaluating and justifying a conclusion on the basis of its putative grounds and warrants. Our research aims to show that, especially in the case of arguments featuring metaphors, ordinary evaluations make use of other sources of reasoning, independent from the argument itself, i.e., actual premises and their connection to the conclusion. Far from being just a source of fallacies, metaphors thus become a cue to creative argumentation.

Challenging the classical view of rationality, the so-called paradigm of bounded rationality (Simon, 1983; Gigerenzer and Selten, 2002; Kahneman, 2003) showed that in most cases humans largely make use of unconscious and automatic intuitions, while reasoning is used merely to offer *post hoc* rationalizations (Tversky and Kahneman, 1981; Evans, 2008; Evans and Frankish, 2009). Reasoning errors do have a psychological dimension (Walton, 2010, p. 159; see also Macagno and Walton, 2010; Godden, 2015), as they are arguments that for some reasons “*seem to be sound* without being so in fact,” where “an argument is sound if it is valid, i.e., it is impossible that its premises are true and its conclusion false, and its premises are true” (Etchemendy, 1990, p. 29). According to this point of view, the *perceived* truth or falsity of the premises represents an important step toward the comprehension of arguments. However, when argumentation exploits the characteristic lexical ambiguity and polysemy of natural language, a robust notion of truth begins to waver. Arguments in natural language often feature loose uses of language, as for instance metaphors, which are “truth independent” (Clark, 1994; Wilson and Sperber, 2002). Previous research shows that the majority of sentences with conventional metaphors are perceived as true, even though they are literally false (Glucksberg, 2001, 2003; Giora, 2003) and the availability of context influences the perception of truth especially in case of novel metaphor (Gildea and Glucksberg, 1983; Glucksberg and Estes, 2000; Bambini et al., 2016). Therefore, the process of interpretation of the premises of a syllogism featuring a metaphor could possibly influence the evaluation of the soundness of the whole argument.

According to the classical pragmatic view (Grice, 1989), sentences featuring a metaphor are considered as literally “patently” false, because of their conventional meaning, and urge for the search of the implicit meaning that better fits the context. Therefore, metaphors might lead to fallacies of reasoning because of meaning ambiguity (Barnden, 2012; Fischer, 2015) and of heuristic rules that never guarantee the preservation of truth (Fischer, 2014; Keefer and Landau, 2016). Indeed, metaphors not only provide arguments with economy of language, greater vividness, interestingness, forcefulness, but also entail a framing effect that implicitly provides a specific perspective to interpret the world. Previous literature claimed that metaphors are a framing strategy through which a (generally more abstract and less known) target conceptual domain is seen in the light of a (generally more concrete and better known) source conceptual domain (Lakoff and Johnson, 1980; Gibbs, 1994; Coulson, 2001; Bowdle and Gentner, 2005). Some relevant properties of the source domain are selected to understand the target domain, but in projecting those properties into the target domain, other properties remain underrated or simply hidden. The mapping of properties from the source to the target implicitly forces the interpreter to consider the target in a specific perspective. Therefore, different metaphorical views might seriously affect one’s reasoning and evaluation of arguments (Lakoff, 2004; Goatly, 2007; Thibodeau and Boroditsky, 2011, 2013; Steen et al., 2014; Semino et al., 2016).

However, precisely these features of metaphors have been said to have elicited a more creative and productive argumentation style, in a number of scientific disciplines ranging from physics and biology (Black, 1962; Hesse, 1963; Kuhn, 1979; Pulaczewska, 2011), to psychology (Holyoak and Thagard, 1995; Indurkhya and Ojha, 2013) and problem solving (Leung et al., 2012; Keefer et al., 2014). Metaphors are indeed based on a cross-domain mechanism of projection (*mapping*), which preserves relations from a source to a target domain, thus favoring analogical reasoning (Gentner et al., 2001; Wolff and Gentner, 2011; Gentner and Asmuth, 2017). In this regard, a metaphor should not be interpreted as a trap leading to fallacies, but as a helpful means to achieve creative thinking (Holyoak and Thagard, 1995; Hofstadter, 1995; Hallyn, 2000; Castro and Marcos, 2011). Far from being just a source of fallacies in reasoning, metaphors might then play a constructive role in reasoning, by enhancing their creative power (Indurkhya, 2007a, 2010). Reconsidering traditional approaches to metaphor as a reasoning device (Black, 1962; Hesse, 1963, 1965; Perelman and Olbrechts-Tyteca, 1969), recent studies claimed that metaphors might be seen as a “condensed analogy” or an “implicit argument” by analogy where some inferences can be drawn from the comparison between the similarities of a source and a target domain (Santibáñez, 2010; Macagno and Zavatta, 2014; Oswald and Rihs, 2014; Svačinova, 2014; Wagemans, 2016).

## Logic and Belief in Reasoning With Metaphors

The similarities between metaphor and analogy have been discussed at both the theoretical and the experimental level. In psychology of reasoning, for instance, Dedre Gentner and her colleagues explicitly stated that “metaphor is like analogy – that the basic processes of analogy are at work in metaphor” (Gentner et al., 2001, p. 243). Like analogies, metaphors are processed as systems of relations, where both the target and the source terms “refer to specific concepts from different semantic domains, and the metaphor is interpreted by aligning the two representations and importing further predicates from the base [source] to the target.” Analogical reasoning thrives on comparisons that are quite frequent in everyday language and play an important role in human reasoning (Vosniadou and Ortony, 1989; Holyoak and Thagard, 1995; Gentner et al., 2001; Shelley, 2003). Metaphors – because of their intrinsic meaning ambiguity – might influence the proper attribution of a certain analogy as a basis for its conclusion. In analogical reasoning featuring a metaphor, it might be argued that a comparison between A and B is established, where A and B share a set X of relevant properties. The relevant properties shared between the target A and the source B of a metaphor are all the properties belonging to the source B that can be mapped onto the target A. Especially in the case of conventional metaphors, the set of relevant properties is given by a “system of associated commonplaces” that usually are assumed to hold true about the source B, applicable to the target and integrated within semantic memory structure (Black, 1954; Gibbs, 1994; Glucksberg, 2003; Bowdle and Gentner, 2005; Kenett et al., 2018). For instance, when we say that “A lawyer is a shark,” we are not claiming that sharks and lawyers share exactly all and the same properties, but rather that a lawyer is comparable to a shark on the basis of a certain set of relevant properties X, including the property of “being aggressive” typically associated with the concept of “shark.” Based on this comparison, if B has the relevant property C belonging to the set X, then it is fair to conclude that A also has the property C. Therefore, given that “A shark is aggressive,” as per analogy, we should conclude that “A lawyer is aggressive”:

(1) [P1] A lawyer is a shark.
  [P2] A shark is aggressive.
  [C] A lawyer is aggressive.

The first premise of the syllogism contains the term “shark,” used with a metaphorical meaning, which also acts as *middle term* of the syllogism, i.e., the term that appears in both the premises of the syllogism, but not in the conclusion and that (properly) connects the premises. The second premise of the syllogism specifies the property on the basis of which the conclusion of the analogical argument is (properly) drawn.

Indeed, a fallacious analogical argument (see Holland et al., 1986 for an extensive account) establishes a faulty analogy as its conclusion, “assuming that it is highly probable there will be some other shared property in a class so wide that there is only a low initial probability of finding any other shared properties relevant to the purpose at hand” (Fearnside and Holther, 1959, p. 4; but see also Bartha, 2010; Macagno et al., 2014, 2017; Macagno, 2017a). In case the property C is not included in the set X of properties belonging to both the target and the source as relevant for the conventional metaphorical meaning, as for instance the literal property of “being a fish,” a faulty analogy drives to the conclusion “A lawyer is a fish”:

(2) [P1] A lawyer is a shark.
  [P2] A shark is a fish.
  [C] A lawyer is a fish.

In such a case, there is a shift in the meaning of the middle term “shark” from the first to the second premise, where the property belonging just to the source “shark” cannot be applied to the target “lawyer.” Therefore, the analogical reasoning is not guaranteed and the argument is fallacious because it contains four terms instead of three terms. In other words, because of the shift in the meaning of the term “shark,” from the metaphorical to the literal one, the argument assumes the structure of a quaternio terminorum (Barth, 1974; Macagno and Walton, 2009; Ervas et al., 2018). Quaternio terminorum is a well known case of a fallacious argument based on the *lexical ambiguity* of its middle term, which assumes different meanings in the two premises (Hamblin, 1970; Woods and Walton, 1989; Copi et al., 2014). If the middle term assumes a different meaning in each premise, then a syllogism, *de facto*, contains a fourth, hidden term, which causes the fallacy. We will call “metaphoric fallacy” a quaternio terminorum based on a lexical ambiguity generated by a metaphor in the first premise of the argument.

Exploiting analogies, metaphors bring together objects belonging to different semantic fields, i.e., lawyers and sharks, which might not have been previously related in any way. From this perspective, metaphors have the power to generate new similarities, triggering certain properties of the source as relevant, even if those same properties were not relevant before. The process of selecting the relevant properties is creative in nature as it gives access to new categorizations (Bowdle and Gentner, 1999, 2005; Glucksberg, 2001, 2008), even though possibly conventionalized by continued usage. For instance, metaphors such as “Lawyers are sharks” can be read as “class-inclusion statements” (Glucksberg and Keysar, 1990; Glucksberg, 2008), if we think of a “shark” as the name of the set whose members display aggressive behavior. Certainly, the statement “Lawyers are sharks” is literally false, if we think of a “shark” as a fish. In this case we are inclined to judge the related argument as fallacious not only because of the irrelevant property “being a fish” in the second premise and/or to the patent literal falsity of the first premise featuring the metaphor, but also because of the patent literal false conclusion: “A lawyer is a fish.” In case of quaternio terminorum with plausible conclusion, the latter effect might be reduced. This fact produces metaphoric fallacies that may appear prima facie strong, i.e., sound arguments with true premises, but actually based on metaphor-related ambiguity of meaning, as in the following examples:

(3) [P1] A lawyer is a shark.
  [P2] A shark takes advantage of others.
  [A] lawyer takes advantage of others.
(4) [P1] A lawyer is a shark.
  [P2] A shark circumvents obstacles.
  [C] A lawyer circumvents obstacles.

The plausible conclusion is given by the fact that the second premise makes a property explicit, which belongs exclusively to the target (“taking advantages from others” in argument 3) and it is not part of the relevant set of X properties shared by both the target and the source, or a property that is not essentially or typically associated to the target nor to the source (“circumventing obstacles” in argument 4) (“emergent properties,” see for instance Gineste et al., 2000; Fauconnier and Turner, 2002; Indurkhya, 2006).

We expected that, in case of a plausible, or at least a conceivable conclusion, the participants might be more careful in evaluating the connection between premises, instead of directly discarding the argument as fallacious on the basis of the patent falsity of its conclusion. In fact, a number of studies on syllogistic reasoning show that the believability of the conclusion strongly influences the evaluation of an argument (Evans et al., 1983; Oakhill et al., 1989; Oakhill and Garnham, 1993; Ball et al., 2006; Correia, 2011). In particular, previous experiments on the “belief-bias” effect in reasoning have emphasized that when presented with deductive arguments to evaluate, participants utter their judgments relying on *a priori* beliefs, rather than on the basis of the arguments themselves. Specifically, irrespective of the actual soundness of the argument, the tendency is to endorse those arguments whose conclusions they believe in and reject those arguments whose conclusions they do not believe in. Some studies (Evans et al., 1983; Correia, 2011) found that, depending on the form of the argument, a conflict between logic and belief might be observed throughout, and at several levels of extent. Other studies (Oakhill et al., 1989; Oakhill and Garnham, 1993; Ball et al., 2006) also showed that beliefs in the conclusion may affect the examination of alternative reasons and act as a filter on putative conclusions.

## Current Research

To the best of our knowledge, however, previous research on argument evaluation interfering with people’s beliefs did not contemplate metaphors in the premises, considering just argument expressed in plain, literal language. Being framing strategies, metaphors might also have a strong influence on the beliefs involved in an argument’s evaluation process. In particular, we expected that the framing effect of metaphor might also depend on the type of metaphor featured in the first premise of the argument. In fact, conventional metaphors are not perceived as metaphors, bearing a status similar to polysemous literal term (Carston, 2002; Giora, 2003; Bowdle and Gentner, 2005; Kenett et al., 2018), and unconsciously act as triggers of “systems of commonplaces” or background of associated beliefs (Black, 1954; Coulson, 2001; Lakoff, 2004; Thibodeau and Boroditsky, 2011, 2013). Novel metaphors, however, are consciously processed as metaphors and require new, original and creative interpretations (Indurkhya, 2007b, 2010; Kenett et al., 2018). We therefore expected a major influence of conventional metaphors in the evaluation of arguments, when compared with novel metaphors, as in the latter case participants are aware of the falsity both of premises and of the deviant, creative interpretation of the conclusion.

Two experimental studies were designed to address the following questions:

(Q1) Are people more prone to commit a faulty analogy/quaternio terminorum fallacy in case of literal terms or in case of metaphors?
(Q2) Does the conventionality of metaphors play any role in case of a metaphoric fallacy?
(Q3) What are the reasons for people to commit a metaphoric fallacy?
(Q4) What is the role of belief in the conclusion in the case of metaphoric fallacy?

The first experiment is designed to answer Q1 and Q2, while the second experiment is aimed to answer Q3 and Q4.

## Experiment 1

In the first experimental study we tested participants’ evaluation of arguments having the standard syllogistic form, comparing literal and metaphorical middle terms. In the case of literal middle terms, their literal meanings in the premises could diverge either because they were *homonymous* (*H*), with two completely different literal meanings, or because they were *polysemous* (*P*), with two partially overlapping literal meanings. In case of metaphorical middle terms, their metaphorical meaning in the first premise could diverge from the literal meaning in the second premise either because they were *conventional metaphors* (*CM*), with the metaphorical meaning already classified in the dictionary, or because they were *novel metaphors* (*NM*), with the metaphorical meaning created from anew.

### Design and Predictions

The experimental design included three sets of arguments (*argument structure condition*) combined with H, P, CM, and NM middle terms (*middle term condition*):

(1) *strong arguments* (6 × H, P, CM, NM middle terms) with true-perceived premises and true-perceived conclusion, where the middle terms were used with the same meaning in the premises;
(2) *standard quaternio terminorum* (6 × H, P, CM, NM middle terms) with true-perceived premises and false-perceived conclusion, where the middle terms were used with different meanings in the premises;
(3) *quaternio terminorum with plausible conclusion* (6 × H, P, CM, NM middle terms) with true-perceived premises and plausible-perceived conclusion, where the middle terms were used with different meanings in the premises.

Fillers included a set of 25 clearly strong arguments and 25 clearly weak arguments. Fillers were designed to check participants’ understanding of the task they were assigned and basic capacity to distinguish between a clearly strong and a clearly weak argument, without any explicit instruction.

Overall, the study had a 3 × 4 experimental design: 3 argument structure conditions × 4 (H, P, CM, NM) middle term conditions. The main effects and possible interaction effect of the two factors (type of argument structure and type of middle term) were planned to be analyzed via a preliminary two-factor ANOVA. A series of paired *t*-test were planned to better understand which conditions had the greatest influence on participants’ evaluation of the arguments. Overall, we had the following main expectations:

(1) A main effect of the argument structure was expected, as the type of conclusion might force the participants to check whether the first and the second premise are connected by a middle term properly used with the same meaning in both the premises. In particular, quaternio terminorum with plausible conclusions was expected to be more difficult to detect when compared to strong arguments, where middle terms need no disambiguation process, and to standard quaternio terminorum, where the patently false conclusion might, *per se*, help participants to detect the fallacy.
(2) A main effect of the middle term was also expected, as the type of middle term might require specific lexical disambiguation process of the meanings in the premises. More specifically, H and NM middle terms were expected to be easier to disambiguate but longer to process in quaternio terminorum as the divergent meanings in the first and in the second premise belong to radically different and not previously associated semantic domains, while P and CM middle terms were expected to be more difficult to disambiguate but shorter to process in quaternio terminorum as the divergent meanings in the first and in the second premise belong to overlapping semantic domains. However, NM middle terms were expected to be more difficult to process when compared to CM middle terms, because novel metaphors are, per definition, unfamiliar, and require wider contexts to be more meaningful and easily understandable, when compared to conventional metaphors, which are lexicalized and very familiar.
(3) A significant interaction between the argument structure and the middle term factors was expected, as a specific lexical disambiguation process of the meanings in the premises is required when the participants needs to check for the possible divergence of meanings in order to evaluate whether the conclusion actually follows from the premises. In particular, we also expected that the framing effect of metaphorical middle terms might interfere with the level of accuracy in the evaluation of arguments.

### Participants

A total of 147 adults (93 women, 54 men, *M*_age_ = 25.03 years, *SD*_age_ = 8.9 years) participated in the experiment. We accepted only those participants whose response to the fillers were correct (acceptance threshold: 90% of correct answers): 19 participants were excluded from the analysis as untrusted participants. All the participants had Italian as their first language and normal/corrected vision. All of them were undergraduate students in Languages and Communication Studies recruited at the University of Cagliari, who did not previously follow any course in logic and/or argumentation theory. The study was approved by the ethics committee of the Department of Education, Psychology, Philosophy at the University of Cagliari and written informed consent was obtained from all participants.

### Materials

The stimulus material consisted of *N* (=122 arguments in Italian, including 50 fillers (see Appendix, **Supplementary Table 10**). To provide the materials for the experiment we previously selected a set of terms (= 206 nouns) that could be used to form H, P, CM, NM middle terms, from the GRADIT (De Mauro, 2000). We devised the arguments on the basis of the middle terms selected from a series of rating studies. In all the arguments with CM and NM middle terms, we made sure that the metaphor appeared in the first premise. We used unambiguous terms to build novel metaphors, checking in the GRADIT that they were not previously used as conventional metaphors.

In case of strong arguments with CM middle terms, we built the second premise following the figurative meaning lexicalized in the GRADIT, in order to have the same (metaphorical) meaning in both the premises and the property in the second premise belonging to the source and applicable to the target. In case of strong arguments with NM middle terms, we followed the same procedure, but we checked that the figurative meaning was not already lexicalized in the GRADIT, and anyway understandable for participants.

In case of standard quaternio terminorum with CM and NM middle terms, we built the second premise following the literal meaning in the GRADIT, in order to have divergent meanings in the premises and the property in the second premise belonging just to the source and not applicable to the target. In case of quaternio terminorum with plausible conclusion with CM and NM middle terms, we built the second premise in the same way but making sure that the conclusion would not appear patently false.

An example in English for each condition is given in **Supplementary Table 1** (see Appendix for the table of all the material in Italian, **Supplementary Table 10**).

### Rating and Pilot Studies

We pre-tested (1) the middle terms, (2) the metaphors in the first premises and (3) the separate premises/conclusions of the arguments in a series of rating studies (*N*_participants_ = 209).

#### Middle Terms

We selected the middle terms according to their *number of letters* and *frequency* (all common terms in the GRADIT, De Mauro, 2000), their *emotional (positive and negative) meaning* and *familiarity* by using a 1 (very negative/very unfamiliar) to 5 (very positive/very familiar) rating scale. We eliminated the terms with definite emotional meanings (*M*_positive meaning_ > 4; *M*_negative meaning_ < 2) and insufficient familiarity (*M*_familiarity_ < 3) (see **Supplementary Table 2**).

#### Metaphors

We tested conventional and novel metaphors along some major psycholinguistic variables (Bambini et al., 2014): *emotional (positive and negative) meaning*, *familiarity*, *meaningfulness* (i.e., confidence in metaphor interpretation) and *comprehension difficulty* by using a 1 (very negative/very unfamiliar/very meaningless/very easy) to 5 (very positive/very familiar/very meaningful/very difficult) rating scale. We eliminated the metaphors with definite emotional meanings (*M*_positive meaning_ > 4; *M*_negative meaning_ < 2), metaphors with insufficient meaningfulness (*M*_meaningfulness_ < 3) and metaphors that were too difficult to understand (*M*_comprehension difficulty_ > 4) (see **Supplementary Table 3**). When compared to conventional metaphors, novel metaphors were rated as less familiar (*p* < 0.001) and more difficult to understand (*p* < 0.01), even though the latter results might have been mitigated by a wider context. Participants also felt less confident in the interpretations they gave to novel metaphors than to conventional metaphors (*p* < 0.01), whose frequent use instead presumably increases participants’ confidence. We did not check for the salience of the properties and properties coherence (Weiland et al., 2014) of the metaphors and this is a limitation that might be particularly relevant when having different figures of the syllogism and thus different directionalities of the argument (Dickstein, 1978; Oberauer et al., 2005) interfering with metaphor directionality. In the present study we just made sure that the same structure of the syllogism was maintained.

#### Premises and Conclusions

We tested the premises of the arguments to make sure that the participants attributed either the same meaning to the middle terms in case of strong arguments or different meanings in case of fallacious arguments. We also tested the premises separately, in order to understand whether they were perceived as either true or false, and the conclusions to understand whether they were perceived as true, false or plausible. We asked participants to verbalize why they perceived premises as false, to avoid false premises that would lead to an “ex falso quodlibet,” and we accepted only those premises that were perceived true. The results showed that 83% of the premises with conventional metaphors were perceived as *true*, while 79% premises with novel metaphors were actually perceived as *false* (Ervas and Ledda, 2014). A pilot study (*N*_participants_ = 40), excluding novel metaphors for this reason, showed a significant effect of conventional metaphors in the evaluation of arguments with plausible conclusion (Ervas et al., 2015). However, we included novel metaphors in the present experiment, because in case of premises with novel metaphors, explanations were offered by participants in order to finally consider them as true premises.

### Procedure

Participants sat in front of a computer in a quiet room. All the participants were tested on Microsoft Windows 7 32-bit Professional Edition. We used PsychoPy 1.81.00 to collect the participants’ answers and response time. The arguments were randomized and visually presented on the computer screen. After gathering initial information about language and education, the participants were asked to read the instructions and complete four practice trials to familiarize them with the task. After presenting the argument on the screen without the middle term, the participants pressed the bar when they were ready to read the middle term (target word), and then the argument disappeared from the screen and the target word appeared for 500 ms on the screen. Then the participants were asked to answer “YES” or “NO” to the following question: “Does the conclusion follow from the premises?”. To evaluate the strength of the arguments, i.e., whether the conclusion [C] follows from the premises [P1] and [P2], we asked the participants a yes/no question by clicking on a “YES” button if they thought that [C] did follow from [P1] and [P2], or a “NO” button otherwise. We registered the “YES”/”NO” answers and the response times from the disappearance of the target word. The overall test lasted for a maximum of 35 min.

### Results

All data were collected at the following address: osf.io/3k27d/. A two-way ANOVA test for accuracy and response times was performed to assess the main effects of the argument structure type and the middle term type and the interaction of the two factors on the evaluation of the arguments (see **Supplementary Table 4**).

A series of paired *t*-tests, corrected for multiple comparisons, were performed to determine the statistical significance. For data analysis, we used the following open source Python packages: sqlalchamy, numpy and scipy. All the results are available at the following address: osf.io/3k27d/. Mean and standard deviation for correct answers and response times are reported in **Supplementary Table 5**.

#### Correct Answers

Overall the results showed a significant main effect of the argument type [*F*_(2,144)_ = 338.3; *p* < 0.01] and the middle term type [*F*_(3,143)_ = 72.10; *p* < 0.01], as well as a significant interaction of the argument type and the middle term type [*F*_(6,140)_ = 61.10; *p* < 0.01] on participant’s evaluation of the arguments (see **Supplementary Table 4**). The significant main effect of the argument type is due to the lower number of correct answers in the case of quaternio terminorum with plausible conclusion condition when compared to both strong argument [*t*_(145)_ = 17.72; *p* < 0.001] and standard quaternio terminorum [*t*_(145)_ = 12.55; *p* < 0.001] conditions. The significant main effect of the middle term type is due to the higher level of accuracy in the case of H middle terms when compared to all the other (P/CM/NM) middle term conditions (*p* < 0.001) and the lower level of accuracy in the case of NM middle terms when compared to all the other (H/P/CM) middle term conditions (*p* < 0.001) (see **Supplementary Table 6**).

Overall, arguments with literal (H and P) middle terms received a significantly higher number of correct answers compared to arguments with metaphorical (CM and NM) middle terms [*t*_(145)_ = 9.11; *p* < 0.001]. In particular, literal (H and P) middle terms received a significantly higher number of correct answers compared to arguments with metaphorical (CM and NM) middle terms in case of strong arguments [*t*_(145)_ = 15.57; *p* < 0.001] and in the case of quaternio terminorum with plausible conclusion [*t*_(145)_ = 6.89; *p* < 0.001], while the difference was not significant in case of standard quaternio terminorum [*t*_(145)_ = −1.39; *p* = 1.01].

Interestingly, in case of strong arguments with CM middle terms, a significantly higher number of participants considered the conclusion as following from the premises, compared to NM middle term condition [*t*_(145)_ = 16.83; *p* < 0.001]. Strong arguments with P literal middle terms received a significantly higher number of correct answers compared to both metaphorical CM middle terms [*t*_(145)_ = 7.87; *p* < 0.001] and NM middle terms [*t*_(145)_ = 25.058; *p* < 0.001] conditions, while in case of standard quaternio terminorum with false conclusion, they received a significantly lower number of correct answers compared to both H literal middle terms condition [*t*_(145)_ = −6.89; *p* < 0.01] and metaphorical CM middle terms [*t*_(145)_ = −4.86; *p* < 0.001] and NM middle terms [*t*_(145)_ = −7.03; *p* < 0.001] conditions. No significant difference was observed between standard quaternio terminorum with CM and NM middle terms (see **Supplementary Table 7**).

In case of quaternio terminorum with plausible conclusion, arguments with H literal middle terms received a significantly higher number of correct answers compared to arguments with metaphorical CM middle terms [*t*_(145)_ = 13.58; *p* < 0.001] and NM middle terms [*t*_(145)_ = 9.98; *p* < 0.001], while arguments with P literal middle terms received a significant higher number of correct answers just in comparison to CM middle terms [*t*_(145)_ = 3.62; *p* < 0.01]. In the case of quaternio terminorum with plausible conclusion, no significant difference was observed between P and NM middle terms, while, interestingly, a significant difference was found between CM and NM middle terms due to the lower number of correct answers in the case of CM middle terms condition [*t*_(145)_ = −3.32; *p* < 0.05) (see **Supplementary Table 7**).

#### Response Time

The response time results for correct answers showed a significant main effect of the argument type [*F*_(2,144)_ = 3.94; *p* = 0.02] and middle term type [*F*_(3,143)_ = 10.56; *p* < 0.01], as well as a significant interaction of the argument type and middle term type [*F*_(6,140)_ = 22.74; *p* < 0.01] (see **Supplementary Table 4**). The significant effect of the argument type is due to the higher response time for the participants’ evaluation of quaternio terminorum with plausible conclusion compared to strong arguments [*t*_(145)_ = 4.07; *p* < 0.001]. The significant effect of the middle term type is due to the higher response time for participants’ evaluation of arguments with NM middle terms [*t*_(145)_ = 3.3; *p* < 0.01] and lower response time for arguments with H middle terms [*t*_(145)_ = −2.98; *p* < 0.05] as compared to the response time registered for the evaluation of arguments with P middle terms (see **Supplementary Table 6**).

Response time analysis also showed that participants took longer to evaluate strong arguments with metaphorical than literal middle terms [*t*_(145)_ = 7.02; *p* < 0.001], they took less time in case of standard quaternio terminorum with metaphorical than literal middle terms [*t*_(145)_ = −3.26; *p* = 0.008]. No significant difference was instead observed in response times when comparing quaternio terminorum with plausible conclusion with metaphorical vs. literal middle terms. In the case of strong arguments with literal middle terms, participants took longer to evaluate arguments with H middle terms as compared to P middle terms [*t*_(145)_ = 12.53; *p* < 0.001] (see **Supplementary Table 6**). In the case of strong arguments with metaphorical middle terms, participants took longer to evaluate arguments with NM middle terms than P middle terms [*t*_(145)_ = 8.57; *p* < 0.001] and H middle terms [*t*_(145)_ = 3.70; *p* < 0.05], but took less time to evaluate arguments with CM middle terms than P middle terms [*t*_(145)_ = −15.1; *p* < 0.001]. In the case of standard quaternio terminorum, higher response time was registered for participants’ evaluation of arguments with P middle terms than H middle terms [*t*_(145)_ = 4.10; *p* < 0.01] and NM middle terms [*t*_(145)_ = 5.57; *p* < 0.001]. In the case of standard quaternio terminorum with metaphorical middle terms, participants took longer to evaluate arguments with CM middle terms compared to P middle terms [*t*_(145)_ = 5.51; *p* < 0.001]. In the case of quaternio terminorum with plausible conclusion, evaluation of arguments with NM middle terms required higher response time compared to both the arguments with P middle terms [*t*_(145)_ = 3.22; *p* < 0.01] and the arguments with CM middle terms [*t*_(145)_ = 1.8; *p* < 0.05] (see **Supplementary Table 8**).

The response time results for wrong answers showed a significant main effect of the argument type [*F*_(2,144)_ = 8.69; *p* < 0.01] and middle term type [*F*_(3,143)_ = 7.10; *p* < 0.01], as well as a significant interaction of the argument type and middle term type [*F*_(6,140)_ = 4.60; *p* < 0.01] (see **Supplementary Table 4**). Response time analysis for wrong answers revealed that higher response time was required for standard quaternio terminorum than quaternio terminorum with plausible conclusion [*t*_(145)_ = 3.03; *p* < 0.05], (see **Supplementary Table 6**). Response time analysis for wrong answers also showed that participants took shorter to evaluate strong arguments with metaphorical than literal middle terms [*t*_(145)_ = −2.66; *p* = 0.05], while no significant difference was observed in response times when comparing quaternio terminorum with metaphorical vs. literal middle terms. In general, higher response time for wrong answers was registered in case of arguments with H middle terms as compared to both arguments with P middle terms [*t*_(145)_ = 3.62; *p* < 0.01] and arguments with CM middle terms [*t*_(145)_ = 3.06; *p* < 0.05] (see **Supplementary Table 6**). In case of strong argument, higher response time for wrong answers was taken just in case of arguments with H middle terms as compared to arguments with P middle terms [*t*_(145)_ = 4.05; *p* < 0.01]. No significant difference was observed for fallacious arguments (standard quaternio terminorum and quaternio terminorum with plausible conclusion).

### Discussion

In Experiment 1, the significant main effect of the argument structure type on the evaluation of the arguments and the comparison among argument structure conditions seem to suggest that quaternio terminorum with plausible conclusion are more difficult to identify compared to both strong arguments, whose middle terms do not require any disambiguation process, and standard quaternio terminorum, whose patently false conclusion might have helped participants in detecting the fallacy. The plausibility of the conclusion might have led participants to consider the quaternio terminorum structure more similar to the structure of a strong argument, thus explaining the higher number of wrong answers and lower response time for wrong answers when compared to the evaluation of standard quaternio terminorum. Moreover, the plausible conclusion might have forced participants to check for the different meanings of the middle term in both the premises, thus explaining the higher response time for participants’ evaluation of quaternio terminorum with plausible conclusion compared to strong arguments where the middle term is used with one and the same meaning.

The significant main effect of the middle term type on participants’ evaluations of the arguments suggests the relevance of the lexical disambiguation process required to understand the possible divergence of the meanings of the middle term in the premises. A disambiguation process, both in the case of literal and metaphorical middle terms, is indeed required to understand whether the premises are properly connected by the same meaning of the middle term in both the premises as in the case of strong arguments, or the premises are improperly connected by a middle term having different meanings in the premises as in the case of quaternio terminorum. Overall, the results support the idea that, overall, literal middle terms are easier to be disambiguated compared to metaphorical middle term, where an inferential step to the metaphorical meaning is required (Grice, 1989). Even in the case of strong argument, where the middle term is used with the same meaning, participants performed worst in the case of metaphorical than literal middle terms and took longer to evaluate strong arguments with metaphorical than literal middle terms. In the case of standard quaternio terminorum, this difference is probably mitigated by the clearly false conclusion, which *per se* might have led participants to detect the fallacy, independently from the (literal or metaphorical) nature of the middle term.

In any case, H middle terms are by far the easiest to be disambiguated, even when compared to literal P middle terms (see **Supplementary Table 6**). Indeed, in the case of H middle term, the different literal meanings are clearly divergent, and the disambiguation process requires a straightforward suppression of one of its two literal meanings, namely the irrelevant one (Gernsbacher, 1990; Gernsbacher and Faust, 1991). In the case of P middle term, there is instead a list of possible, partially overlapping meanings that might be selected (Gick and Holyoak, 1983; Gentner et al., 1993). Higher response time for H middle term condition than for P middle term condition suggests that, in the case of H middle terms, two completely different meanings need to be processed, while in the case of P middle terms just divergent properties of the same meaning need to be processed to evaluate the argument. Indeed, even though in both H disambiguation and P interpretation a suppression process is required (Gernsbacher and Faust, 1991; Gernsbacher et al., 2001; Rubio Fernandez, 2007), in P the meanings of the middle term share some semantic properties as they overlap, while in H the suppressed meaning has no semantic relation with the contextually relevant one.

In the case of metaphorical middle term, the literal and the non-literal meanings possibly share some properties, which allow metaphorical understanding. Also in the case of metaphors, the properties of the literal linguistically encoded concept are active in the early phases of lexical access (Weiland et al., 2014), when some properties of the linguistically encoded concept are selected to understand the communicated (“*ad hoc*”) concept (Glucksberg et al., 2001; Rubio Fernandez, 2007). In lexical pragmatics (Carston, 2002; Recanati, 2004, 2010), a process of modulation (narrowing or broadening) has been proposed to explain the selection of the relevant meaning in both P and CM, where the concept communicated by a term has more restricted (lexical narrowing) or more general (lexical broadening) interpretation than the linguistically encoded concept. For instance, to understand the metaphor “Lawyers are sharks,” we select the properties of the literal, linguistically encoded concept “shark” that are required to grasp the *ad hoc* concept, i.e., being aggressive, engaged in constant struggle, etc. Other irrelevant properties of the linguistically encoded concept “shark,” as for instance “being a fish,” are suppressed. This process is particularly crucial when assessing arguments with P and CM middle terms, where the property made explicit in the second premise might belong to the set of (ir)relevant properties of the middle term that (im)properly connect the premises of the argument. This process might therefore be a source of mistakes, when not carefully controlled, in evaluating whether the conclusion follows from the premises, and might explain the shorter response times for P and CM middle terms in the case of wrong answers compared to H middle terms (see **Supplementary Table 6**).

However, the results also show that the participants performed better when evaluating strong arguments with P middle terms than metaphorical (CM and NM) middle terms, but worst when evaluating standard quaternio terminorum with P middle terms than metaphorical (CM and NM) middle terms. These results suggest that strong arguments with metaphorical middle terms are more difficult to evaluate than arguments with literal P middle terms, because metaphors in the first premise act anyway as framing strategies influencing the reading of the overall argument. This would also explain why, in the case of wrong answers, participants took less time to evaluate strong argument with metaphorical than literal middle terms: they did not realize the framing effect of metaphors, implicitly influencing their reading of the overall argument. On the contrary, the patently false conclusion of standard quaternio terminorum might be more easily attributed by participants to the presence of metaphors in the first premises, altering the perception of the truth conditions and to the irrelevant literal property made explicit in the second premise, not applicable to the target in the conclusion of the argument. Instead, when evaluating a quaternio terminorum with plausible conclusion, participants overall performed worse than both the case of strong arguments and standard quaternio terminorum and especially in the case of metaphorical than literal middle terms, suggesting that the framing effect of metaphors might be particularly strong and alter participants’ beliefs in the reading of the argument with plausible conclusion. Therefore, these results suggest that the answer to Q1 – “Are people more prone to commit a faulty analogy/quaternio terminorum fallacy in case of literal terms or in case of metaphors?” – is the following R1:

R1: People are more prone to commit a quaternio terminorum fallacy in the case of syllogisms with plausible conclusion compared to syllogisms with false conclusion. In the case of plausible conclusion, people are more prone to fall into the fallacy when middle term is a metaphor (metaphoric fallacy) rather than a literal term.

The metaphorical NM middle term condition is anyway the most difficult compared to both the literal and the metaphorical CM middle term conditions (see **Supplementary Table 6**). This could be due to the fact that, as the results of the rating studies showed, novel metaphors were more unfamiliar, difficult to interpret and perceived as less meaningful in a narrow context compared to conventional metaphors: participants needed to think more and offer further explanations to justify the premises as true. The narrow context of the syllogism makes it more difficult to interpret novel metaphors in the first premise and to assess whether the property made explicit in the second premise might be properly attributed to the target: the availability of a wider context would have helped participants to make sense not only of the NM middle term but also of the overall argument featuring a novel metaphor (Gildea and Glucksberg, 1983; Glucksberg and Estes, 2000; Lai et al., 2009). This would explain the significant lower number of correct answers in the case of strong argument with NM middle terms compared to literal (H and P) and CM middle terms. Instead, in the case of standard quaternio terminorum, the “patent falsity” of the novel metaphor and the conclusion might have helped the participants in discarding the argument as fallacious. This would explain the higher number of correct answers and the shorter response time in the case of standard quaternio terminorum with NM middle terms as compared to P middle terms. Compared to response times required to disambiguate H middle terms in the case of quaternio terminorum, no significant difference with response times required to disambiguate NM middle terms would be justified by the fact that in both cases two completely divergent meanings need to be processed. Interestingly, standard quaternio terminorum with CM middle terms required a longer response time to be evaluated as compared to P middle terms, probably because the covert framing effect and the set of properties stereotypically associated with the conventional metaphor need to be inhibited in the second premise to evaluate a property not applicable to the metaphor target.

In any case, the previous literature coming from different theoretical approaches agrees on the fact that novel metaphor comprehension is more demanding in terms of contextual and encyclopedic knowledge (Glucksberg and Estes, 2000; Glucksberg, 2003; Giora, 2003; Bambini et al., 2016; Kenett et al., 2018), as a completely creative meaning, divergent from the literal one, is intended. In particular, when evaluating quaternio terminorum with plausible conclusion, participants might have taken a longer time in the case of NM middle terms as compared to P and CM middle terms, because of the search of a creative meaning of NM that is able to make sense of the plausible conclusion. Quaternio terminorum with plausible conclusion featuring a novel metaphor were easier to detect than those featuring a conventional metaphor, even though they took longer to process. The interpretation process of novel metaphors diverges from that of conventional metaphors, as the literal meaning would not be suppressed and might endure eliciting conscious communicated affective and imagistic effects (Indurkhya, 2007b, 2016; Thibodeau and Durgin, 2008; Carston, 2010). While conventional metaphors are processed faster as they activate a tacit system of commonplaces including the relevant properties associated with the conventional meaning of the metaphor, novel metaphors took longer to process because participants were aware of the fact that a completely new, creative meaning, divergent from the literal one had to be generated in the argumentation. Therefore, the results suggest that the answer to Q2 – “Does the conventionality of metaphors play any role in case of a metaphoric fallacy?” – is the following R2:

R2: CM middle terms are the most reliable predictor of the metaphoric fallacy with plausible conclusion compared to NM middle terms. It is probably because arguments with NM middle terms are consciously processed as leading to new and creative metaphorical interpretations.

## Experiment 2

In the second experiment, our goal was to understand *why* participants were more prone to accept a quaternio terminorum with plausible conclusion as sound, especially in the case of a metaphoric fallacy. We therefore tested how different factors (understandability, convincingness, emotional appeal, logical relation, ambiguity, belief in the conclusion, real world experience) contribute to the participants’ evaluation of arguments with plausible conclusion, comparing literal (H and P) and metaphorical (CM and NM) middle terms conditions.

### Design and Predictions

The study had a 1 × 4 experimental design: 1 argument structure condition (quaternio terminorum with plausible conclusion) × 4 (H, P, CM, NM) middle term conditions. The detection of the ambiguity of the meanings of the middle term in the premises was expected to be the most important reason to answer that the conclusion did not follow from the premises independently of the middle term condition, thus evaluating the quaternio terminorum – even though with a plausible conclusion – as a fallacious argument based on lexical ambiguity. We also expected that the perceived understandability of the argument and perceived logical relation between premises and conclusion might influence the participants’ evaluation of the argument soundness.

We did not expect participants to find the arguments emotionally appealing as we explicitly avoided the middle terms with definite emotional meanings. We did instead expect that the believability of the conclusion would strongly influence the evaluation of the arguments, as previous literature testified (Evans et al., 1983; Oakhill et al., 1989; Oakhill and Garnham, 1993; Ball et al., 2006; Correia, 2011). The belief in the conclusion was expected to be most rated in the CM middle term condition, because of the covert metaphorical framing effect influencing participants’ beliefs.

### Participants

Fifty participants (31 women, 8 men, 1 other, *M*_age_ = 26.62 years, *SD*_age_ = 4.88 years) were recruited from the University of Cagliari for the experiment. All of them had Italian as their first language and did not previously follow courses in logic and/or argumentation theory. The study was approved by the ethics committee of the Department of Education, Psychology, Philosophy at the University of Cagliari and written informed consent was obtained from all the participants.

### Materials

The stimulus material for Experiment 2 consisted of 48 arguments in Italian, composed by a subset of the materials used in Experiment 1, i.e., the set of N = 24 quaternio terminorum with plausible conclusion combined with 6 × H, P, CM, NM middle terms (see **Supplementary Table 10** in Appendix, column “Quaternio terminorum with plausible conclusion,” for the subset of materials in Italian used in Experiment 2), and N = 24 fillers as clearly strong arguments without lexical ambiguous middle terms.

### Procedure

To conduct the experiment, an online Google form was created. The arguments were randomly shown to participants. After gathering initial information about language and education, participants were asked the yes/no question on whether the conclusion of the given argument followed from the premises, as in the first experiment. Then they were asked the following questions related to understandability, convincingness, emotional appeal, logical relation, ambiguity, belief in the conclusion and real world experience:

Understandability: *Do you understand the argument?*
Convincingness: *Is the argument convincing in anyway?*
Emotional appeal: *Is the argument emotionally appealing?*
Logical relation: *Is the conclusion logically related to premises?*
Ambiguity: *Is the ambiguity at any level influencing?*
Belief in the conclusion: *Do you believe in C (independent of P1 and P2)?*
Real world experience: *Do you have any experience of similar arguments?*

Participants were asked to rate the arguments for each question on the scale of 1–5 (1 being least likely and 5 being most likely). The average time to finish the experiment was 30 min.

### Results

A linear regression analysis was performed on the data. Separate linear models were created for 4 conditions (H, P, CM, and NM) in both “yes” and “no responses.” Results of the second experiment confirmed the first experiment results as to what concerns the answers to the yes/no question on whether the conclusion follows from the premises [H (Yes = 83.75%; No = 16.25%)], [P (Yes = 91.75%; No = 8.25%)], [CM (Yes = 82.5%; No = 17.5%)], [NM (Yes = 85%; No = 15%)]. When the conclusion was perceived to be following from the premises, the results showed that, for any middle term type, understandability and belief in the conclusion were significant predictors for committing the fallacy of quaternio terminorum [Understandability: H (*t*_(48)_ = 3.7; *p* < 0.001), P (*t*_(48)_ = 3.15; *p* < 0.01), CM (*t*_(48)_ = 2.85; *p* < 0.01), and NM (*t*_(48)_ = 2.65; *p* < 0.001); Belief in the conclusion: H (*t*_(48)_ = 3.7; *p*<0.001), P (*t*_(48)_ = 3.42; *p* < 0.01), CM (t_(48)_ = 3.9; *p* < 0.05), and NM (t_(48)_ = 3.6; *p* < 0.01)]. Logical relation was a significant predictor in the case of H [*t*_(48)_ = 3.65; *p* < 0.01] and CM [*t*_(48)_ = 3.9; *p* < 0.01] middle terms conditions. However, ambiguity was a significant predictor for recogniszing the fallacy of quaternio terminorum when the conclusion was seen not to be following from the premises in P [*t*_(48)_ = 3.58; *p* < 0.01], CM [*t*_(48)_ = 4.7; *p* < 0.001], and NM [*t*_(48)_ = 3.2; *p* < 0.01] middle terms conditions. Understandability [*t*_(48)_ = 4.1; *p* < 0.01] and real world experience [*t*_(48)_ = 3.26; *p* < 0.001] were instead significant predictor just in H middle term condition (see **Supplementary Table 9**).

### Discussion

The results show that when people commit the fallacy of quaternio terminorum, independently from the middle term type, they think to have understood the argument and they believe in the conclusion of the (fallacious) argument, independent from its premises. Just in the case of H middle terms and CM middle terms, they also think to have found a logical relation. While in the case of fallacious arguments with H middle terms participants believing in the conclusion might simply have searched for a possible (logical) connection between the two meanings of the middle term to justify their answer, in the case of fallacious arguments with CM middle terms, the covert framing effect of the conventional metaphor might have played a major role in both believing in the conclusion and thinking of having found a logical relation. Indeed, the framing effect of the conventional metaphor and the property made explicit in the second premise might have forced participants to look at the metaphor target under a certain perspective, influencing the overall reading of the argument and its believability. In this sense, conventional metaphors alter participants’ perception of the strength of the metaphoric fallacy, making it appear strong. Interestingly, when participants detected the fallacy of quaternio terminorum with H middle terms they did not think of ambiguity as the main cause, even though quaternio terminorum is per definition the fallacy of lexical ambiguity. Participants instead recognized ambiguity as the main source of the fallacy in the case of quaternio terminorum with metaphorical (and especially CM) middle terms. The results therefore suggest that the answer to Q3 – “What are the reasons for people to commit a metaphoric fallacy?” – is the following R3:

R3: The most prominent reasons for committing a metaphoric fallacy is the understandability of the argument and the participants’ belief in the conclusion of the argument, independent of its premises. The most prominent reason for detecting the metaphoric fallacy is ambiguity.

Previous literature on the effect of the belief in the conclusion in the evaluation of literal arguments have already shown that there is a conflict between logic and belief in syllogistic reasoning: people are more likely to endorse arguments whose conclusion appears believable (Evans et al., 1983; Ball et al., 2006; Correia, 2011). It also showed that, especially in the case of a believable conclusion, which is not in contrast with the premises, participants tend to feel justified in confirming their own belief in the conclusion, even though it does not follow from the premises. Logic should be accepted in spite of the believability of the conclusion, but participants tend to read the premises and make sense of the overall argument by confirming their own (prior) beliefs concerning the conclusion (Baron, 1988; Kunda, 1999).

In the second experiment, we investigated if the belief bias influenced metaphorical arguments as well. When comparing the results of the second experiment on the reasons why participants considered the metaphoric fallacy as a strong argument, in both CM and NM middle term conditions, participants indicated to have understood the argument and to firmly believe in the conclusion independent of its premises. However, only in the case of CM middle terms they indicated to have found a logical relation. In this regard, novel metaphors seem to be less persuasive compared to conventional metaphors. This might be due to the fact that, in the narrow argumentative context, conventional metaphors are subconsciously perceived as true, while novel metaphors are known to elicit creative interpretations that depart from the conventional, literal meanings. Moreover, in the case of NM middle terms, participants were aware from the very beginning that their own beliefs play a major role in the creative interpretation of the premises and thus on the evaluation of the overall argument. Indeed, they did not claim to have found a logical relation between the premises and the conclusion.

However, in CM middle terms condition, participants were unaware of the metaphorical framing effect in the first premise and probably reassessed the overall argument on the basis of their conscious belief in the conclusion. Thus, in the case of arguments with believable conclusion, participants may have reinterpreted the premises of the arguments with conventional metaphors in order to make sense of the believed conclusion and pretending to have found the proper logical connection between the conclusion and the premises of the argument. In this process, it seems that the conventional metaphor is revitalized as it is no more interpreted with its conventional meaning, but with an alternative, new and creative meaning that is able to justify the believed conclusion. The new reading of the metaphor in the first premise is extended to the conclusion through the second premise in order to make the conclusion follow from the premises. The second premise makes a property explicit, which is not part of the relevant set of properties commonly associated with the metaphor and shared by the source and the target, but that might be plausibly mapped onto the target in the conclusion of the argument. The arguments with a plausible conclusion and CM middle terms are thus interpreted metaphorically on the whole. Therefore, the answer to Q4 – “What is the role of belief in the conclusion in the case of metaphoric fallacy?” – might be the following R4:

R4: The belief in the conclusion leads participants to reinterpret the overall argument, finding alternative reasons to make the premises consistent with the believed conclusion. Especially in the case of CM middle terms, a process of revitalization of the metaphor seems to be required to extend the new metaphorical interpretation to the overall argument.

## General Discussion and Conclusion

The main findings of our experiment are that the type of argument structure and the type of middle term influence participants’ evaluation of the arguments, the metaphoric fallacy included. Indeed, the results suggest that the structure of quaternio terminorum with plausible conclusion is by far the most difficult to evaluate compared to both the strong argument, where the middle term is used with the same meaning in both the premises and the standard quaternio terminorum structure, where the patently false conclusion facilitates the detection of the fallacy. The results also suggest that arguments’ evaluation depends on the specific lexical disambiguation process of the middle term meanings in the premises: in general, literal middle terms (and especially H middle terms) made it easier to evaluate an argument than metaphorical middle terms (especially NM middle terms). In the case of quaternio terminorum, H middle terms are easier to disambiguate even when compared to P middle terms, because the disambiguation process deals with two completely different meanings and thus with no possible overlapping properties, while literal P middle terms are the most difficult to evaluate because the disambiguation process involves specific properties of the same semantic domain, which have to been evaluated and compared with the property made explicit in the second premise. A similar process of disambiguation is in place in the case of CM middle terms, even though, in the case of standard quaternio terminorum, the patent falsity of the conclusion might be more easily attributed to the presence of a metaphor in the first premise and to the patently irrelevant property (not stereotypically associated with the conventional metaphor) in the second premise. On the contrary, in quaternio terminorum with plausible conclusion condition, participants performed better in P middle terms rather than CM middle terms condition, whose covert framing effect might have further influenced participants’ evaluation.

In the case of the metaphoric fallacy, the plausibility of the conclusion might have led participants to search for alternative interpretation of the metaphor in the first premise with longer response times, especially in the case of NM middle terms. However, the metaphoric fallacy with plausible conclusion tends to be evaluated as a strong argument especially when the metaphor is conventional rather than novel. Diversely from creative metaphors, conventional metaphors are not neutral with respect to the participants’ beliefs as they entail a framing effect associated with a system of commonplaces usually held to be true and covertly activated (Lakoff, 2004; Thibodeau and Boroditsky, 2011, 2013). When the analogy settled via a CM middle term leads to a faulty, but plausible conclusion, there might arise “a conflict between two types of thought processes, one logical reasoning according to the instructions and the other a response on the basis of their prior beliefs” (Evans, 2004, p. 139–140). The main finding of the second experiment is that this conflict is at work in the case of metaphoric fallacy with plausible conclusion, where the participants’ belief in the conclusion might force them to search for alternative reasons to connect the believed conclusion and the premises. This process might be implicit in the case of CM middle terms, where participants also believed to have found a logical relation, and might lead to a creative revitalization of conventional metaphors (Goldstein et al., 2012) and generation of alternative interpretations in the light of the believed conclusion.

Overall, the experiments suggest that while novel metaphors consciously lead participants toward creative interpretations from the very beginning, i.e., when they read the first premise featuring the metaphor, conventional metaphors covertly influence their reading of the argument, especially when the conclusion is believable. In this sense, arguments with believable conclusion featuring CM middle terms are more persuasive than arguments featuring NM middle terms. As it happens in many cases of biases inducing to fallacies (Correia, 2011), the participants believing in the conclusion are unaware of committing to a faulty analogy and creatively searching for alternative reasons to adjust the interpretation of the premises to align them with the conclusion. The revitalization of the conventional metaphor CM and the creative interpretation of the premises are therefore guided by the need to confirm participants’ belief in the conclusion.

The literature on “confirmation bias,” i.e., the tendency to favor information confirming one’s own beliefs, is quite vast (see Oswald and Grosjean, 2004 for a review), and started with Bacon’s “Novum Organum” (1620: XLVI), where he stated that “the human understanding when it has once adopted an opinion (either as being the received opinion or as being agreeable to itself) draws all things else to support and agree with it”. Far from being irrational, people are motivated to preserve their own beliefs and they are able to find alternative reasons to maintain them, postponing the actual logical relation between premises and conclusion. Especially when this phenomenon occurs unintentionally, the interpretation of the premises seems to come from a process of selective evidence collection required to confirm participants’ (prior) beliefs on the conclusion part (Baron, 1988; Oakhill et al., 1989; Kunda, 1990; Ball et al., 2006). In the case of syllogistic reasoning featuring a metaphor in the first premise, the main source of evidence to maintain the conclusion is the second premise, making explicit the property of the source to be mapped onto the target. The property might either belong to the set of (literal) properties of the source not applicable to the target, or to the set of (metaphorical) properties shared by the source and the target, as they have been associated with the conventional meaning of the metaphor. In the first case, a patently false conclusion should derive and be easily recognized, while in the second case a true conclusion should be derived from the use of the same metaphorical meaning in both the premises.

It is possible to speculate that plausible conclusions in metaphoric fallacies might come from premises making explicit either a property belonging to the set of (literal) properties of the target, or to the set of “emergent properties” of the metaphor, i.e., properties that are not associated with neither the source nor the target (Gineste et al., 2000; Wilson and Carston, 2006). For instance, when we say that someone is a “bulldozer” or a “block of ice,” the relevant property of “being insensitive” or “being reserved” do not belong, respectively, to the source concept of “bulldozer” or “block of ice,” but “emerge” from the metaphorical use of those words. Especially in the case of novel metaphors, the emergence of properties is not directly connected to the source and/or shared by the source and the target, but might be linked either to a conceptual combination of the target and the source domains based on the encyclopedic knowledge about them (Glucksberg and Estes, 2000; Wilson and Carston, 2006; Vega Moreno, 2007), or to the images evoked by the source and the target concepts mentioned in the metaphor (Davidson, 1978; Indurkhya, 2006, 2007b, 2016; Carston, 2010). We can hypothesize that similar processes are activated in the revitalization of conventional metaphors for the creative search of reasons to confirm the conclusion. The presence of narrow argumentative context given by the second premise affects the process of revitalization in two main ways. First, it explicitly alters the relevant information that might be included in the source concept, which in turn affects the contextual assumptions and implications of the metaphor. Second, it puts forward certain goals, expectations or even imagined scenarios in the evaluator of arguments with a metaphorical middle term. The inferences that can be drawn from certain goals, expectations and imagined scenarios are a form of backward inference from an expected conclusion to the premises needed to derive it (Wilson and Carston, 2006; Mazzone, 2015). Therefore, in the accidental comparison by analogy, i.e., based on properties that essentially neither belong to the metaphor nor to the literal source, the compared concepts are contextually redefined (Indurkhya, 1992; Vega Moreno, 2007; Goldstein et al., 2012; Macagno et al., 2014). This process of modulation and adjustment of the premises in order to derive the believed conclusion leads to the revitalization of the metaphor even in a narrow argumentative context, such as the one presented to the participants of the experiments, and solicit a more creative style of reasoning when compared to the conventional use of a metaphor.

## Future Directions

The results presented in this article raise various interesting questions on the creative role of metaphors in reasoning. An aspect that needs further examination is related to the effect of the figure of the syllogism on the evaluation of arguments (Dickstein, 1978; Oberauer et al., 2005) as the order of the words influences the sequential reading and interpretation of the argument. This is particularly interesting in the case of metaphors, whose directionality effect (Black, 1954, 1962; Tversky, 1977; Goodblatt and Glicksohn, 2017; Indurkhya and Ojha, 2017) might interfere with the directionality of the syllogistic figure used. In this perspective, data on the salience of the properties, the kind of properties (of the target/of the source/shared/emergent) (Gineste et al., 2000) and properties coherence (Weiland et al., 2014) of metaphors should definitely be taken into account. Another interesting aspect that can be investigated is related to the effect of the emotional meaning of the middle terms on the evaluation of the arguments. In the present study we selected middle terms with “emotionally neutral” meaning. However, emotions have been shown to act as framing strategies that influence reasoning (De Sousa, 1987; Damasio, 1994; Frijda et al., 2000), it would be interesting to investigate whether and to what extent the detection of the metaphoric fallacy is influenced by the presence of a metaphor based on an “emotive word” (Stevenson, 1944; Macagno and Walton, 2010; Macagno, 2017a), i.e., positive- or negative-valenced word.

A possible limitation of the study regards its ecological validity, even though the results are potentially interesting to further research in specific contexts or real-life settings. For instance, the contextual information of an argument might be too narrow to produce the typical imagistic effect novel metaphors possess (Carston, 2010; Indurkhya, 2016), and a wider context might also influence participants’ perception of metaphors as true and/or awareness of the entailed framing effect (Thibodeau and Boroditsky, 2011, 2013; Kövecses, 2015). Presenting a wider context where the argument is inserted, participants might come up with more and varied emergent properties of the metaphor as well as with more creative solutions to make the premises fit the believed conclusion. Further research is therefore required to shed a light on the mechanisms of the creative use of metaphors in argumentation.

## Ethics Statement

This study was carried out in accordance with the recommendations of the ethics committee of the University of Cagliari (Department of Pedagogy, Psychology, Philosophy) consent from all subjects. All subjects gave written informed consent in accordance with the Declaration of Helsinki. The protocol was approved by the ethics committee of the Department of Pedagogy, Psychology, Philosophy, University of Cagliari.

## Author Contributions

FE, AL, AO, and BI: experimental design. FE and AL: construction of the materials and rating studies. FE, AL, AO, and GP: data collection. GP: data analysis and programming of the 1st experiment. AO: data analysis and programming of the 2nd experiment. FE: manuscript writing. All the authors provided feedback on the draft and approved the final version of the manuscript.

## Conflict of Interest Statement

The authors declare that the research was conducted in the absence of any commercial or financial relationships that could be construed as a potential conflict of interest.
