# Supplementary material for: Creative Argumentation: When and Why People Commit the Metaphoric Fallacy
Source: Front Psychol. 2018 Sep 25;9:1815. doi: 10.3389/fpsyg.2018.01815 (PMC6168032; doi:10.3389/fpsyg.2018.01815)
Supplement: Supplementary file 1 [file Table_1.DOCX]

***Supplementary Material***

Creative Argumentation:
When and Why People Commit the Metaphoric Fallacy

**Francesca Ervas*, Antonio Ledda, Amitash Ojha, Giuseppe Antonio Pierro, Bipin Indurkhya**

*** Correspondence:** Francesca Ervas: [francesca.ervas@gmail.com](mailto:francesca.ervas@gmail.com)

# Supplementary Data

All data, results and code program were collected at the following publicly available address: [osf.io/3k27d/](osf.io/3k27d).

# Tables

**Table 1**. Examples in English for each argument structure condition combined with H/P/CM/NM middle term conditions

|  | **Strong arguments** | **Standard quaternio terminorum** | **Quaternio terminorum with plausible conclusion** |
| --- | --- | --- | --- |
| **H** | [P1] Barclays is a *bank*.  [P2] A *bank* is a financial institution.  [C] Barclays is a financial institution. | [P1] Barclays is a *bank*.  [P2] A *bank* is a riverside.  [C] Barclays is a riverside. | [P1] Barclays is a *bank*.  [P2] A *bank* is at river’s edge.  [C] Barclays is at river’s edge. |
| **P** | [P1] An alphabet character is a *letter*.  [P2] A *letter* is a symbol.  [C] An alphabet character is a symbol. | [P1] An alphabet character is a *letter*.  [P2] A *letter* is sent in an envelope.  [C] An alphabet character is sent in an envelope. | [P1] An alphabet character is a *letter*.  [P2] A *letter* is a written communication.  [C] An alphabet symbol is a written communication. |
| **CM** | [P1] Life is a *puzzle*.  [P2] A *puzzle* is complex.  [C] Life is complex. | [P1] Life is a *puzzle*.  [P2] A *puzzle* is a toy.  [C] Life is a toy. | [P1] Life is a *puzzle*.  [P2] A *puzzle* is made of pieces.  [C] Life is made of pieces. |
| **NM** | [P1] Old age is a *dinner*.  [P2] A *dinner* is at the end of the days.  [C] Old age is at the end of the days. | [P1] Old age is a *dinner*.  [P2] A *dinner* is a meal.  [C] Old age is a meal. | [P1] Old age is a *dinner*.  [P2] A *dinner* is quite long.  [C] Old age is quite long. |

**Table 2**. Mean (M) and standard deviation (SD) for each measure of H/P/CM/NM middle terms

| **Middle terms** | **Frequency** | | **Number of letters** | | **Emotional meaning** | | | **Familiarity** | |
| --- | --- | --- | --- | --- | --- | --- | --- | --- | --- |
|  | M | SD | M | SD | M |  | SD | M | SD |
| **H** | 4.69 | 2.92 | 6.39 | 1.16 | 3.17 | | .61 | 3.98 | .39 |
| **P** | 4.9 | 3.73 | 6.5 | 1.07 | 3.40 | | .47 | 4.20 | .25 |
| **CM** | 5.31 | 3.88 | 6.67 | 1.10 | 3.45 | | .39 | 4.16 | .18 |
| **NM** | 4.09 | 3.70 | 6.39 | 1.01 | 3.43 | | .27 | 4.15 | .26 |

**Table 3**. Mean (M) and standard deviation (SD) for each measure of the metaphors

| **Metaphors** | **Emotional** **meaning** | | **Familiarity** | | **Meaningfulness** | | **Comprehension** **difficulty** | |
| --- | --- | --- | --- | --- | --- | --- | --- | --- |
|  | M | SD | M | SD | M | SD | M | SD |
| **CM** | 3.49 | .49 | 3.85 | .78 | 4.08 | .47 | 1.90 | .72 |
| **NM** | 3.33 | .60 | 1.85 | .44 | 3.39 | .38 | 2.95 | .58 |

**Table 4**. Main effects of Argument type and Middle term type for accuracy and response times

|  | **Accuracy** | | | | **Response time**  **(correct answers)** | | | | **Response time**  **(wrong answers)** | | | |
| --- | --- | --- | --- | --- | --- | --- | --- | --- | --- | --- | --- | --- |
|  | SS | df | F | p | SS | df | F | p | SS | df | F | p |
| **C(Argument)** | 34.98 | 2 | 338.3 | < .01 | 20.69 | 2 | 3.94 | .02 | 180.52 | 2 | 8.69 | < .01 |
| **C(Middle term)** | 11.17 | 3 | 72.10 | < .01 | 82.79 | 3 | 10.56 | < .01 | 221.19 | 3 | 7.10 | < .01 |
| **C(Argument): C(Middle term)** | 18.95 | 6 | 61.10 | < .01 | 356.61 | 6 | 22.74 | < .01 | 286.97 | 6 | 4.60 | < .01 |

Sum of squares (SS), degrees of freedom (df)

**Table 5**. Mean (M) and standard deviation (SD) values of correct answers/response times for argument structure conditions, combined with H/P/CM/NM middle terms

| **Argument structure condition** | | **H** | | **P** | | **CM** | | **NM** | |
| --- | --- | --- | --- | --- | --- | --- | --- | --- | --- |
|  |  | M | SD | M | SD | M | SD | M | SD |
| **Strong arguments** | correct answers | .92 | .27 | .95 | .22 | .83 | .37 | .46 | .50 |
|  | response time | 2.32 | 2.94 | .94 | 1.41 | 2.54 | 2.79 | 2.53 | 3.02 |
| **Standard quaternio terminorum** | correct answers | .83 | .38 | .68 | .47 | .71 | .41 | .83 | .38 |
|  | response time | 2.17 | 2.95 | 2.92 | 4.59 | 1.78 | 2.2 | 1.99 | 2.53 |
| **Quaternio terminorum with plausible conclusion** | correct answers | .67 | .47 | .44 | .50 | .35 | .48 | .43 | .50 |
|  | response time | 2.67 | 3.58 | 1.84 | 2.05 | 2.05 | 2.59 | 2.88 | 3.26 |

**Table 6**. t/p values for accuracy and response times for correct and wrong answers, comparing argument structure and middle term conditions

|  | **Comparisons** | **Accuracy** | **Response time**  **(correct answers)** | **Response time**  **(wrong answers)** |
| --- | --- | --- | --- | --- |
| **Argument structure  conditions** | Strong arguments/Standard quaternio terminorum | .49 | -1.7 | -1.52 |
|  | Strong arguments/Quaternio terminorum with PC | 17.72*** | -4.07*** | 1.94 |
|  | Standard quaternio terminorum/Quaternio terminorum with PC | 12.55*** | -2.38 | 3.03* |
| **Middle term  conditions** | H/P middle terms | 6.3*** | 2.98* | 3.62** |
|  | H/CM middle terms | 8.36*** | 1.01 | 3.06* |
|  | H/NM middle terms | 14.84*** | -0.52 | 2.54 |
|  | P/CM middle terms | 1.9 | -2.1 | - .63 |
|  | P/NM middle terms | 7.48*** | -3.3** | -1.92 |
|  | CM/NM middle terms | 5.5*** | -1.48 | -1.05 |

PC = plausible conclusion; *p < .05, **p < .01, ***p < .001

**Table 7**. t/p values for correct answers for argument structure conditions, comparing H/P/CM/NM middle term conditions

| **Argument structure condition** | **H/P** | **H/CM** | **H/NM** | **P/CM** | **P/NM** | **CM/NM** |
| --- | --- | --- | --- | --- | --- | --- |
| Strong arguments | -2.62 | 5.37*** | 22.84*** | 7.87*** | 25.58*** | 16.83*** |
| Standard quaternio terminorum | 6.89** | 5.23 | .131 | -4.86*** | -7.03*** | -2.14 |
| Quaternio terminorum with plausible conclusion | - .37 | 13.58*** | 9.98*** | 3.62** | .3 | -3.32* |

*p < .05, **p < .01, ***p < .001

**Table 8**. t/p values for response times for argument structure conditions, comparing middle term conditions

| **Argument structure condition** | **H/P** | **H/CM** | **H/NM** | **P/CM** | **P/NM** | **CM/NM** |
| --- | --- | --- | --- | --- | --- | --- |
| Strong arguments | 12.53*** | 1.84 | -3.70* | 15.1*** | -8.57*** | -2.28 |
| Standard quaternio terminorum | -4.10** | -2.78 | 2.70 | -5.51*** | 5.57*** | -.13 |
| Quaternio terminorum with plausible conclusion | 1.99 | 1.66 | -1.53 | .16 | -3.22** | -1.8* |

*p < .05, **p < .01, ***p < .001

**Table 9**. t/p values for each predictor in the evaluation of arguments with plausible conclusion, comparing H/P/CM/NM middle terms conditions

| **Condition** | **Predictors** | **H** | | | **P** | | | | **CM** | | | **NM** | | | |
| --- | --- | --- | --- | --- | --- | --- | --- | --- | --- | --- | --- | --- | --- | --- | --- |
|  |  | β | t | R^2^ | β | t | R^2^ | | β | t | R^2^ | β | t | R^2^ | |
| **YES** |  |  |  | .62 |  |  | .73 | |  |  | .77 |  |  | .56 | |
|  | Understandability | .14 | 3.7*** |  | .02 | 3.15*** | |  | .17 | 2.85** |  | .04 | 2.65*** | |  |
|  | Convincingness | .11 | 1.41 |  | .07 | 2.76 |  | | .08 | 1.29 |  | .12 | 2.11 |  | |
|  | Emotional appeal | .19 | 2.7 |  | .14 | 1.46 |  | | .15 | 1.25 |  | .07 | 1.6 |  | |
|  | Logical relation | .09 | 3.65** |  | .08 | 1.71 |  | | .03 | 3.9** |  | .07 | 2.6 |  | |
|  | Ambiguity | .02 | 2.25 |  | .15 | 2.17 |  | | .05 | 2.67 |  | .03 | 1.12 |  | |
|  | Belief on the conclusion | .09 | 3.7*** |  | .08 | 3.42** |  | | .15 | 3.9* |  | .11 | 3.6** |  | |
|  | Real world experience | .07 | 2.6 |  | .03 | 1.96 |  | | .03 | 1.13 |  | .15 | 1.9 |  | |
|  |  |  |  |  |  |  |  | |  |  |  |  |  |  | |
| **NO** |  |  |  | .32 |  |  | .15 | |  |  | .51 |  |  | .49 | |
|  | Understandability | .05 | 4.1** |  | .11 | 2.36 |  | | .02 | 1.46 |  | .14 | 2.2 |  | |
|  | Convincingness | .02 | 1.2 |  | .16 | 1.12 |  | | .07 | 1.31 |  | .06 | 2.4 |  | |
|  | Emotional appeal | .05 | 2.61 |  | .09 | 2.45 |  | | .01 | 1.33 |  | .05 | 1.7 |  | |
|  | Logical relation | .12 | 1.5 |  | .02 | 2.11 |  | | .11 | 2.42 |  | .11 | 1.5 |  | |
|  | Ambiguity | .18 | 1.55 |  | .07 | 3.58** |  | | .17 | 4.7*** |  | .07 | 3.2** |  | |
|  | Belief on the conclusion | .09 | 2.1 |  | .11 | 1.77 |  | | .10 | 0.97 |  | .09 | 2.1 |  | |
|  | Real world experience | .02 | 3.26*** |  | .09 | 1.2 |  | | .08 | 2.01 |  | .13 | 1.71 |  | |

*p < .05, **p < .01, ***p < .001

**Table 10**. Table of materials in Italian for each argument structure condition combined with H/P/CM/NM middle term conditions

|  | **Strong arguments** | **Standard quaternio terminorum** | **Quaternio terminorum with plausible conclusion** |
| --- | --- | --- | --- |
| **H** | [P1] Il caseificio produce grana.  [P2] Grana è formaggio.  [C] Il caseificio produce formaggio.  [P1] La televisione è un media.  [P2] Un media è molto diffuso.  [C] La televisione è molto diffusa.  [P1] La zia coltiva il miglio.  [P2] Il miglio è una pianta.  [C] La zia coltiva una pianta.  [P1] Al bivio c’è un blocco.  [P2] Un blocco è un’interruzione.  [C] Al bivio c’è un’interruzione.  [P1] Belen è una valletta.  [P2] Una valletta è una showgirl.  [C] Belen è una showgirl.  [P1] In montagna c’è una scarpata.  [P2] Una scarpata è un piano inclinato.  [C] In montagna c’è un piano inclinato. | [P1] La prugna contiene il nocciolo.  [P2] Il nocciolo è un albero.  [C] La prugna contiene un albero.  [P1] Una certa quantità è un tasso.  [P2] Un tasso scava buche.  [C] Una certa quantità scava buche.  [P1] Fabrizio Corona è un narciso.  [P2] Un narciso è un fiore.  [C] Fabrizio Corona è un fiore.  [P1] I capelli hanno un riccio.  [P2] Un riccio è un porcospino.  [C] I capelli hanno un porcospino.  [P1] Un capitombolo è un rotolone.  [P2] Un rotolone è un nastro arrotolato.  [C] Un capitombolo è un nastro arrotolato.  [P1] La buccia è uno scarto.  [P2] Uno scarto è una grossa differenza.  [C] La buccia è una grossa differenza. | [P1] Una scommessa è una puntata.  [P2] Una puntata è trasmessa in TV.  [C] Una scommessa è trasmessa in TV.  [P1] Un assortimento è una gamma.  [P2] Una gamma è greca.  Un assortimento è greco.  [P1] Un portiere ha fatto una parata.  [P2] Una parata è una sfilata militare.  [C] Un portiere ha fatto una sfilata militare.  [P1] La guardia forestale ha una mazzetta.  [P2] Una mazzetta è una somma illegale.  [C] La guardia forestale ha una somma illegale.  [P1] A Verona c’è l’arena.  [P2] L’arena è sabbia.  [C] A Verona c’è sabbia.  [P1] Talete è un saggio.  [P2] Un saggio è una lettura piacevole.  [C] Talete è una lettura piacevole. |
| **P** | [P1] Arisa incide un album.  [P2] Un album è una raccolta di brani.  [C] Arisa incide una raccolta di brani.  [P1] Anna Frank ha scritto un diario.  [P2] Un diario contiene pensieri.  [C] Anna Frank ha scritto pensieri.  [P1] Una nave utilizza una banchina.  [P2] Una banchina è un molo del porto.  [C] Una nave utilizza un molo del porto.  [P1] Un viaggiatore ha una mappa.  [P2] Una mappa è una carta geografica.  [C] Un viaggiatore ha una carta geografica.  [P1] Il giudice decretò il rinvio.  [P2] Il rinvio è una proroga.  [C] Il giudice decretò una proroga.  [P1] Il compositore scrive una melodia.  [P2] Una melodia è una composizione musicale.  [C] Il compositore scrive una composizione musicale. | [P1] Un lingotto è una barra.  [P2] Una barra è una lineetta.  [C] Un lingotto è una lineetta.  [P1] La luna forma uno spicchio.  [P2] Uno spicchio è una parte del frutto.  [C] La luna forma una parte del frutto.  [P1] Il nuotatore percorre una corsia.  [P2] Una corsia è una sezione stradale.  [C] Il nuotatore percorre una sezione stradale.  [P1] Una piantina è una cartina.  [P2] Una cartina avvolge una sigaretta.  [C] Una piantina avvolge una sigaretta.  [P1] Una coperta imbottita è un piumino.  [P2] Un piumino è un giubbotto.  [C] Una coperta imbottita è un giubbotto.  [P1] Gli ecologisti lanciano un appello.  [P2] Un appello è una data d’esame.  [C] Gli ecologisti lanciano una data d’esame. | [P1] Quella trasmissione è una replica.  [P2] Una replica contrasta il suo libro.  [C] Quella trasmissione contrasta il suo libro.  [P1] Mario incide una cassetta.  [P2] Una cassetta è un contenitore.  [C] Mario incide un contenitore.  [P1] L’occhio ha l’iride.  [P2] L’iride è un insieme di colori.  [C] L’occhio ha un insieme di colori.  [P1] La benzina ha avuto un rialzo.  [P2] Un rialzo è un rilievo.  [C] La benzina ha avuto un rilievo.  [P1] Carla aggiunge una bustina.  [P2] Una bustina è un piccolo involucro.  [C] Carla aggiunge un piccolo involucro.  [P1] Il papa ha un seggio.  [P2] Un seggio ha un elettorato.  [C] Il papa ha un elettorato. |
| **CM** | [P1] Mario segue il gregge.  [P2] Il gregge è un gruppo conformista.  [C] Mario segue un gruppo conformista.  [P1] L’aggressività è un’armatura.  [P2] Un’armatura è una difesa.  [C] L’aggressività è una difesa.  [P1] Gigi guadagna la pagnotta.  [P2] La pagnotta è il sostentamento quotidiano.  [C] Gigi guadagna il sostentamento quotidiano.  [P1] La vita è un rebus.  [P2] Un rebus è complesso.  [C] La vita è complessa.  [P1] I valori costituiscono uno sfondo.  [P2] Uno sfondo è un sistema di riferimento.  [C] I valori costituiscono un sistema di riferimento.  [P1] L’amicizia è un supporto.  [P2] Un supporto è un aiuto.  [C] L’amicizia è un aiuto. | [P1] Studiare è una maratona.  [P2] Una maratona è una corsa podistica.  [C] Studiare è una corsa podistica.  [P1] La mamma è una chioccia.  [P2] La chioccia cova le uova.  [C] La mamma cova le uova.  [P1] I soci fondatori sono il nucleo.  [P2] Il nucleo è all’interno dell’atomo.  [C] I soci fondatori sono all’interno dell’atomo.  [P1] L’amicizia è un balsamo.  [P2] Un balsamo è una preparazione emolliente.  [C] L’amicizia è una preparazione emolliente.  [P1] L’attività ha visto un decollo.  [P2] Un decollo è una manovra dell’aereo.  [C] L’attività ha visto una manovra dell’aereo.  [P1] Il comico è una sagoma.  [P2] Una sagoma è una linea di contorno.  [C] Il comico è una linea di contorno. | [P1] Franco ha chiuso un capitolo.  [P2] Un capitolo è una parte del libro.  [C] Franco ha chiuso una parte del libro.  [P1] Quella ragazza è una gemma.  [P2] Una gemma è una pietra preziosa.  [C] Quella ragazza è una pietra preziosa.  [P1] Una serie di eventi è un’ondata.  [P2] Un’ondata è uno tzunami.  [C] Una serie di eventi è uno tzunami.  [P1] La presidenza è l’apice.  [P2] L’apice è la parte più alta.  [C] La presidenza è la parte più alta.  [P1] Una risata è un farmaco.  [P2] Un farmaco serve alla salute.  [C] Una risata serve alla salute.  [P1] Il corpo è un tempio.  [P2] Il tempio è sacro.  [C] Il corpo è sacro. |
| **NM** | [P1] Il migliore amico è un sosia.  [P2] Un sosia è una persona somigliante.  [C] Il migliore amico è una persona somigliante.  [P1] L’altruismo è una rincorsa.  [P2] Una rincorsa è uno slancio.  [C] L’altruismo è uno slancio.  [P1] La mafia è un polpo.  [P2] Un polpo ha tentacoli.  [C] La mafia ha tentacoli.  [P1] Un fan è uno sponsor.  [P2] Uno sponsor favorisce l’organizzazione.  [C] Un fan favorisce l’organizzazione.  [P1] L’odio è una steppa.  [P2] Una steppa è arida.  [C] L’odio è arido.  [P1] L’onore è una casata.  [P2] Una casata è la difesa dell’identità.  [C] L’onore è la difesa dell’identità. | [P1] Un vortice è un cannolo.  [P2] Un cannolo è siciliano.  [C] Un vortice è siciliano.  [P1] Un’esperienza è un camper.  [P2] Un camper ha 4 ruote.  [C] Un’esperienza ha 4 ruote.  [P1] Una cozza è un astuccio.  [P2] Un astuccio contiene penne.  [C] Una cozza contiene penne.    [P1] Il movimento è una canoa.  [P2] La canoa ha una pagaia.  [C] Il movimento ha una pagaia.  [P1] Il vento è un bidello.  [P2] Un bidello è un uomo.  [C] Il vento è un uomo.  [P1] L’Africa è un arazzo.  [P2] Un arazzo decora le pareti.  [C] L’Africa decora le pareti. | [P1] Il gallo è un faraone.  [P2] Un faraone è un capo.  [C] Il gallo è un capo.  [P1] Quel bimbo è un pandoro.  [P2] Il pandoro è dolce.  [C] Quel bimbo è dolce.  [P1] Un grido è un megafono.  [P2] Un megafono emette suoni.  [C] Un grido emette suoni.  [P1] Uno spirito è uno gnomo.  [P2] Uno gnomo è un personaggio di fantasia.  [C] Uno spirito è un personaggio di fantasia.  [P1] Il cuore è un’anfora.  [P2] Un’anfora trasporta liquidi.  [C] Il cuore trasporta liquidi.  [P1] Il lavoro è un badile.  [P2] Un badile serve nei campi.  [C] Il lavoro serve nei campi. |
